# Modulation of the wheat transcriptome by TaZFP13D under well-watered and drought conditions

**DOI:** 10.1007/s11103-023-01403-y

**Published:** 2024-02-09

**Authors:** William Bouard, François Ouellet, Mario Houde

**Affiliations:** Département des Sciences biologiques, Université du Québec à Montréal, Montréal, QC H3C 3P8 Canada

**Keywords:** Drought tolerance, *Triticum aestivum*, Barley stripe mosaic virus, C2H2 zinc finger protein, Oxidative stress, Chloroplast

## Abstract

Maintaining global food security in the context of climate changes will be an important challenge in the next century. Improving abiotic stress tolerance of major crops such as wheat can contribute to this goal. This can be achieved by the identification of the genes involved and their use to develop tools for breeding programs aiming to generate better adapted cultivars. Recently, we identified the wheat *TaZFP13D* gene encoding Zinc Finger Protein 13D as a new gene improving water-stress tolerance. The current work analyzes the *TaZFP13D*-dependent transcriptome modifications that occur in well-watered and dehydration conditions to better understand its function during normal growth and during drought. Plants that overexpress *TaZFP13D* have a higher biomass under well-watered conditions, indicating a positive effect of the protein on growth. Survival rate and stress recovery after a severe drought stress are improved compared to wild-type plants. The latter is likely due the higher activity of key antioxidant enzymes and concomitant reduction of drought-induced oxidative damage. Conversely, down-regulation of *TaZFP13D* decreases drought tolerance and protection against drought-induced oxidative damage. RNA-Seq transcriptome analysis identified many genes regulated by TaZFP13D that are known to improve drought tolerance. The analysis also revealed several genes involved in the photosynthetic electron transfer chain known to improve photosynthetic efficiency and chloroplast protection against drought-induced ROS damage. This study highlights the important role of TaZFP13D in wheat drought tolerance, contributes to unravel the complex regulation governed by TaZFPs, and suggests that it could be a promising marker to select wheat cultivars with higher drought tolerance.

**Supplementary Information:**

The online version contains supplementary material available at 10.1007/s11103-023-01403-y.

## Introduction

Global population is constantly growing, currently exceeding 8 billion people and expected to reach 10 billion by 2050 (United Nations Department of Economic and Social Affairs 2022). Consequently, food demand is expected to increase by approximately 50% in 2050 (Van Dijk et al. 2021) resulting in a need to increase crop yields. However, environmental changes associated with global warming have increased drought spells frequency and severity, which intensifies water scarcity in croplands and contributes to yield loss (Dai 2013; Zampieri et al. 2017). Increasing the yield of the most cultivated and consumed food crops, like wheat, will play a crucial role to address the coming food security challenges.

In response to water-stress, plants accumulate the phytohormone ABA, which is a key player in growth regulation and stress responses (Kamiyama et al. 2021). Physiological responses to drought stress involve complex ABA-dependent and ABA-independent signal perception and transduction networks. ABA mediates stomatal closure (Munemasa et al. 2015), which is crucial to limit water loss by transpiration. Closing stomata improves water retention at the expense of CO_2_ influx in leaves (Flexas et al. 2006). This limits the carboxylation activity of ribulose-1,5-bisphosphate carboxylase/oxygenase (Rubisco) and favors photorespiration. In this context, plants suffering drought episodes continue to harvest light, while photosynthetic electron transport chain (PETC) products (ATP and NADPH) are less consumed by the Calvin-Benson-Bassham cycle, leading to PETC over-reduction. PETC saturation exacerbates ROS production at multiple sites, within the PETC, and in other organelles (Cruz de Carvalho 2008; Foyer and Hanke 2022; Khorobrykh et al. 2020; Sasi et al. 2018; Vijayaraghavareddy et al. 2022). Excess electron flow is redirected to alternative electron sinks like the water-water cycle (Mehler reaction) or photorespiration (Asada 1999; Biehler and Fock 1996; Noctor et al. 2002). This leads to increased chloroplastic and peroxisomal ROS production, requiring effective ROS scavenging protection systems to prevent oxidative damages to proteins, lipids, or nucleic acids, which could negatively impact growth, yield, and threaten the plant’s survival (Cruz de Carvalho 2008; Gururani et al. 2015; Noctor et al. 2002). The wheat photosynthetic apparatus is therefore often damaged during drought stress. Unsurprisingly, drought tolerant wheat cultivars show better photosystem protection compared to less tolerant cultivars (Liu et al. 2006).

Studies conducted in Arabidopsis revealed the important role of the C1-2i C2H2 zinc-finger protein (ZFP) family in abiotic stress tolerance, including drought and oxidative stress (Liu et al. 2022; Xie et al. 2019). In addition, members of this ZFP subfamily have been associated with drought tolerance in monocot species like rice (Huang et al. 2009; Li et al. 2021; Yuan et al. 2018; Zhang et al. 2014). A previous work from our laboratory identified the C1-2i *TaZFPs* family in wheat, and expression profile analysis revealed that this transcription factor family is stress-responsive (Cheuk and Houde 2016). The most up-regulated *TaZFP* in response to all the stress tested, *TaZFP1B*, was shown to be an important positive regulator of drought tolerance (Cheuk et al. 2020). Recently, we have selected five other candidate *TaZFPs* based on their expression profile in response to abiotic stress, and evaluated the drought stress tolerance of wheat seedlings overexpressing each candidate gene (Bouard and Houde 2022). This selection identified *TaZFP13D* as a new positive regulator of drought stress tolerance. In the present study, we focus on the molecular characterization of this gene and its corresponding protein. Identification of genes and pathways regulated by TaZFP13D will help to better understand its function within the *TaZFP* family and in drought tolerance. Our results indicate that many genes regulated by TaZFP13D are known to improve drought tolerance and chloroplast protection against drought/oxidative stress-induced oxidative damage.

## Materials and methods

### Plant material and growth conditions

*Nicotiana benthamiana* and wheat (*Triticum aestivum* cultivar Atlas66, obtained from Carver and colleagues (Carver et al. 1993) seedlings were grown in E15 Conviron growth cabinets at 22 °C and 70% relative humidity, under fluorescent and incandescent lighting (photon flux density of 100 µmol m^−2^ s^−1^) with a 16 h/8 h (day/night) photoperiod. *N. benthamiana* and wheat plants were cultivated in a mixture of black earth, perlite, peat moss (2:1:1, v:v:v) and watered with 20:20:20 N:P:K (0.5 g L^−1^). Control wheat plants (named well-watered) were watered daily up to 35 days (as indicated in the figure legends). For drought treatment, plants were first watered daily for 14 days (Feekes growth stage 1), then water was withheld for different periods (as indicated in the figure legends).

### Subcellular localization

To determine the subcellular localization of TaZFP1B and TaZFP13D, two chimeric fluorescent fusion constructs were generated by PCR overlap extension (See Online Resource 1 for primers). The *TaZFP1B* coding sequence was fused to the mCherry sequence, while *TaZFP13D* was fused to the eYFP sequence. A 4-residue glycine hinge was added between the *TaZFP* coding sequence and the fluorescent reporter. The resulting DNA fragments were PCR amplified and cloned in the pAVA321 vector (von Arnim et al. 1998) after removal of the green fluorescent protein coding sequence. Control constructs for expression of only mCherry or YFP were also generated. The four constructs (named 35S::mCherry, 35S::TaZFP1B-mCherry, 35S::eYFP, 35S::TaZFP13D-eYFP) were cloned into the pPZP121 binary vector (Hajdukiewicz et al. 1994). The four vectors were transformed into *Agrobacterium* EHA105 and the strains were used to infiltrate 4 to 5-week-old *N. benthamiana* leaves as described previously (Cheuk and Houde 2017). Leaf samples were harvested two days after infiltration and then vacuum infiltrated for 2 min with Hoechst 33342 (1 µg mL^–1^), 0.2% (v/v) Triton X-100 to stain nuclei. Samples were rinsed thoroughly with distilled water to remove excess dye before analysis by fluorescence imaging as previously described (Bouard and Houde 2022).

### Generation of wheat plants with modified *TaZFP13D* expression

The four-component Barley Stripe Mosaic Virus (BSMV) expression system (Cheuk and Houde 2018) was used to modulate *TaZFP13D* expression levels in the wheat cultivar Atlas66 as previously described (Bouard and Houde 2022). Briefly, this vector is based on four different viral RNAs (α, β, γa and γb), which are encoded by the respective pCaBS plasmids (Fig. 2a and b). A sequence of interest can be inserted within the γa and/or γb viral RNA by ligation independent cloning (LIC). The previously generated pCaBS-γ1-mCherry plasmid was used in all experiments since it allows the monitoring of plant infection by confocal microscopy (Cheuk and Houde 2018). To overexpress or down-regulate *TaZFP13D*, we used the previously generated pCaBS-γ2-TaZFP13D and pCaBS-γ2-siRNA13D plasmids (Genbank accession number of *TaZFP13D*: OM630429) (Bouard and Houde 2022). All plasmids were transformed into *Agrobacterium tumefaciens* EHA105, then mixtures of the pCaBS-α, pCaBS-β, pCaBS-γ1-mCherry strains with either the pCaBS-γ2 (for empty vector), pCaBS-γ2-TaZFP13D (OEX) or pCaBS-γ2-siRNA13D (siRNA) strain were agroinfiltrated in *N. benthamiana* leaves to produce BSMV extracts. Soluble extracts of *N. benthamiana* were prepared and used to inoculate wheat seedlings as previously described (Bouard and Houde 2022; Cheuk and Houde 2017). After inoculation, wheat seedlings were potted in soil mix at a density of 10 plants per pot (13.5–13.5–13 cm; length-width-height). Since the BSMV approach is a system that does not generate transgenic lines stable over multiple generations, new plants must be inoculated every time a new experiment is designed, and for each replicate.

### Determination of growth parameters

For biomass determination, aerial parts of well-watered or drought-treated plants were weighed before and after drying at 70 °C for 72 h. To minimize the effect of variation between individual plants, aerial parts from 10 different plants were pooled together to constitute one biological replicate. Values obtained are means +/− standard deviation (SD) from at least three biological replicates. The survival rate (%) was determined on 14-day-old well-watered seedlings that were drought-treated for 14 days then allowed to recover by re-watering for 7 days.

### Antioxidant enzyme activity

Tissue samples from at least three different second leaves of wheat seedlings were used to measure superoxide dismutase (SOD), ascorbate peroxidase (APX) and catalase (CAT) enzyme activities as previously described (Bouard and Houde 2022).

### Determination of lipid peroxidation

Oxidative damage to lipids was evaluated on leaf samples harvested from well-watered or drought-treated plants (see figure legend for stress duration). Each sample (0.5 g) was composed of three different wheat second leaves and malondialdehyde content was determined by the thiobarbituric acid reactive substance (TBARS) test as described previously (Cheuk et al. 2020).

### RNA extraction and qRT-PCR analyses

Four types of plants (Wild-type, empty vector, BSMV TaZFP13D OEX and BSMV TaZFP13D siRNA) were grown under well-watered conditions for 21 days (well-watered) or for 14 days before withholding water for 7 days (drought). Tissue samples composed of second leaves from three different plants were harvested at the same time of day and flash frozen in liquid nitrogen. Total RNA was extracted using the Monarch® Total RNA Miniprep Kit (New England Biolabs). RNA reverse transcription, qRT-PCR analysis, and fold-change calculation were performed as described previously (Bouard and Houde 2022). See Online Resource 1 for primers.

### RNA-seq libraries preparation, sequencing, and analysis

Twelve RNA samples were used for RNA-Seq, with each plant type grown in well-watered (6 RNA samples) or drought conditions (6 RNA samples): two replicates from control plants (one replicate from WT and one from empty vector), two replicates from OEX and two replicates from siRNA plants. The empty vector sample was used to discriminate the potential impact of the BSMV infection on gene expression in the OEX and siRNA plants. Quality control of total RNA samples, RNA-Seq libraries preparation and paired-end sequencing (2 × 150 bases) were performed at Novogene (California, USA) using the Illumina NovaSeq platform. Raw data provided by Novogene (available on GEO repository, #GSE226842) were processed and analyzed using the web-based platform Galaxy (http://usegalaxy.org). Reports of the raw reads quality were obtained with the FastQC tool (version 0.11.9) (Andrews 2010). To improve the quality of reads, we used the wrapper script Trim-galore (version 0.6.3) to remove sequencing adapters (Illumina universal) and four additional bases at the 5’ and 3’ ends of each read, retained reads with a Phred quality score exceeding 30, and discarded reads shorter than 20 nucleotides. Sequencing artifacts were filtered from the remaining reads using the Fastx_toolkit (version 0.0.14) and reads quality improvement was verified with the FastQC tool (version 0.11.9). *Triticum aestivum* reference transcriptome (release 52) was retrieved from Ensembl Plants (https://plants.ensembl.org) and used for transcripts abundance quantification using Salmon (version 1.5.1) in quasi-mapping mode, with Kmer size set to 31 (Patro et al. 2017). Transcripts-levels count tables obtained with Salmon and the *Triticum aestivum* gff3-type annotation file (release 52 from Ensembl Plants) were then used to determine the differentially expressed genes (DEGs) in TaZFP13D OEX and TaZFP13DsiRNA samples with the DESeq2 software (version 2.11.40.7) (Love et al. 2014). We have selected DEGs with a Log 2-fold change ≥ 1.5 or ≤ − 1.5 and an adjusted p-value (FDR) < 0.001, compared to all other types of plants cultivated under the same growth condition. For example, 3 analyses were run for well-watered OEX plants: OEX to WT, OEX to empty vector, and OEX to siRNA. Only the DEGs respecting the cut-off in all three analyses were retained. Protein sequences of the selected DEGs were retrieved using the Biomart tool of Ensembl Plants and used to determine gene functional annotation using NCBI BLAST tool. Based on gene annotation, selected DEGs were categorized in different classes (Online Resource 2). Genes-level normalized count files provided by the DESeq2 software were then used to produce gene expression heatmaps using the web server Heatmapper (Babicki et al. 2016).

To compare transcriptome modifications mediated by TaZFP1B and TaZFP13D, we used mRNA libraries previously generated during the characterization of TaZFP1B (available on GEO repository, #GSE136683), using the same wheat cultivar and experimental design as the present study (Cheuk et al. 2020). This dataset was used to identify DEGs in TaZFP1B OEX and siRNA plants as described above. However, due to the reduced number of biological replicates of this dataset, we used more stringent cut-offs (Log 2-fold change ≥ 2.3 or ≤ − 2.3 for OEX and ≥ 4 or ≤ − 4 for siRNA, with adjusted p-value (FDR) < 0.0001) to retain a similar DEGs number compared to TaZFP13D DEGs selection.

## Results

### Subcellular localization of TaZFP13D and TaZFP1B

**Fig. 1:**
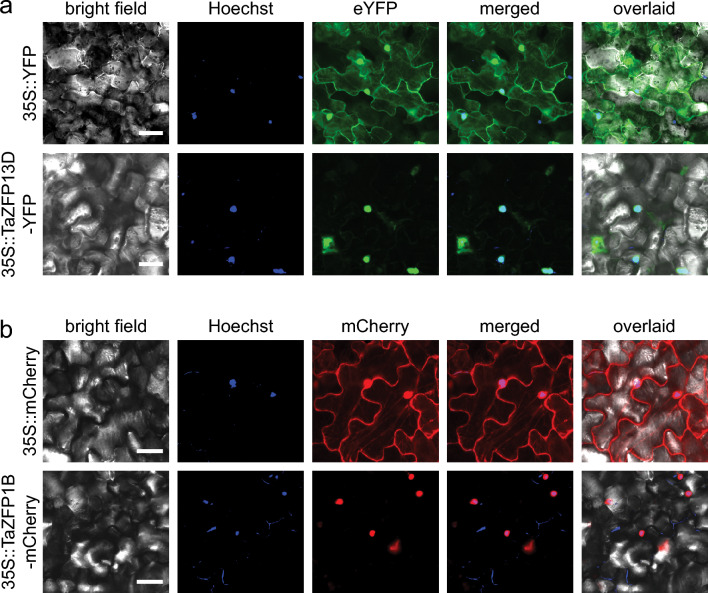
TaZFP13D and TaZFP1B subcellular localization. The TaZFP13D-eYFP chimeric protein or eYFP (**a**) and TaZFP1B-mCherry chimeric protein or mCherry (**b**) were transiently expressed by Agroinfection of *N. benthamiana* leaves. Fluorescence was captured by confocal imaging 2 days after infiltration, and nuclei were stained with Hoechst 33342. Scale bars represent 50 μm.

Among the TaZFP family, TaZFP13D and TaZFP1B have been associated with drought tolerance in wheat (Bouard and Houde 2022; Cheuk et al. 2020). Given the prediction of a Nuclear Localization Signal (NLS) motif in each amino acid sequence (Bouard and Houde 2022), these two ZFPs are expected to be translocated to the nucleus. To confirm this, confocal microscopy was used to determine the subcellular localization of TaZFP13D::eYFP (Fig. 1a) and TaZFP1B::mCherry (Fig. 1b) fusion proteins in Agro-infiltrated *N. benthamiana* leaves. While the eYFP and mCherry controls showed diffuse expression throughout the cells, the chimeric TaZFP13D-eYFP and TaZFP1B-mCherry proteins were restricted to the nuclei, as confirmed by Hoechst 33342 staining. These results show that TaZFP13D and TaZFP1B are properly targeted to nuclei, which supports their putative function as nuclear transcription regulators.

### TaZFP13D improves growth and drought tolerance

**Fig. 2:**
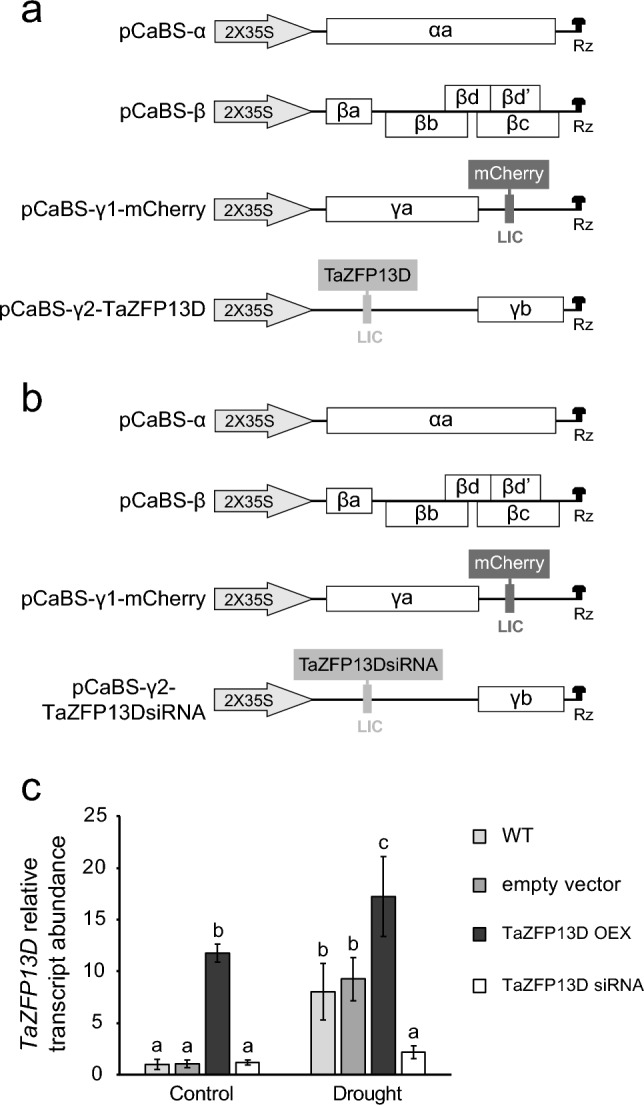
Modulation of *TaZFP13D* expression in the wheat cultivar Atlas66**. **Schematic representation of the four BSMV vectors used to prepare *N. benthamiana* soluble extracts for *TaZFP13D* overexpression (OEX) (**a**) and down-regulation (siRNA) (**b**) in the wheat line Atlas66. As infection control, plants were infected with viruses produced from the same vectors except that an empty pCaBS-γ2 vector (no sequence inserted in the LIC cloning site) was used (empty vector). Rz: ribozyme sequence; LIC: ligation independent cloning site. (**c**) The non-infected (WT) and empty-vector BSMV-infected plants were grown in well-watered condition for 21 days (Control), or for 14 days followed by a drought treatment performed by withholding water for 7 days (Drought). *TaZFP13D* expression levels were measured by qRT-PCR. Data are means +/− S.D. of three biological replicates, and statistical analysis was performed by one-way ANOVA followed by a Tuckey’s test (P < 0.05). Different letters indicate a statistical difference. See Online Resource 1 for primers.

In a previous work, we used the four component BSMV system to overexpress (OEX) or down-regulate (siRNA) *TaZFP13D* expression in the wheat cultivar Atlas66 (Bouard and Houde 2022). The same vectors (Fig. 2a and b) and infection method were used to inoculate Atlas66 seeds for all experiments performed in the present study. To confirm that *TaZFP13D* expression level is modulated in BSMV-infected plants, *TaZFP13D* transcript abundance was quantified by qRT-PCR (Fig. 2c). As expected, a 7-day drought treatment of control plants (WT and empty vector plants) at the seedling stage increased the expression of the endogenous *TaZFP13D* gene by approximately 8-fold. Well-watered OEX plants showed a strong overexpression of *TaZFP13D* (11-fold increase) compared to well-watered control plants. In OEX plants, in which *TaZFP13D* transcript level is already high, drought treatment increased *TaZFP13D* expression by only 1.5-fold compared to OEX in well-watered condition. This difference in drought-induced up-regulation of *TaZFP13D* expression between WT and OEX plants (8X vs. 1.5X) suggests that a maximum threshold level of transcripts is reached. In contrast, silencing prevented accumulation of *TaZFP13D* transcripts after drought treatment, confirming that the siRNA efficiently targets the mRNA encoding this gene.

**Fig. 3:**
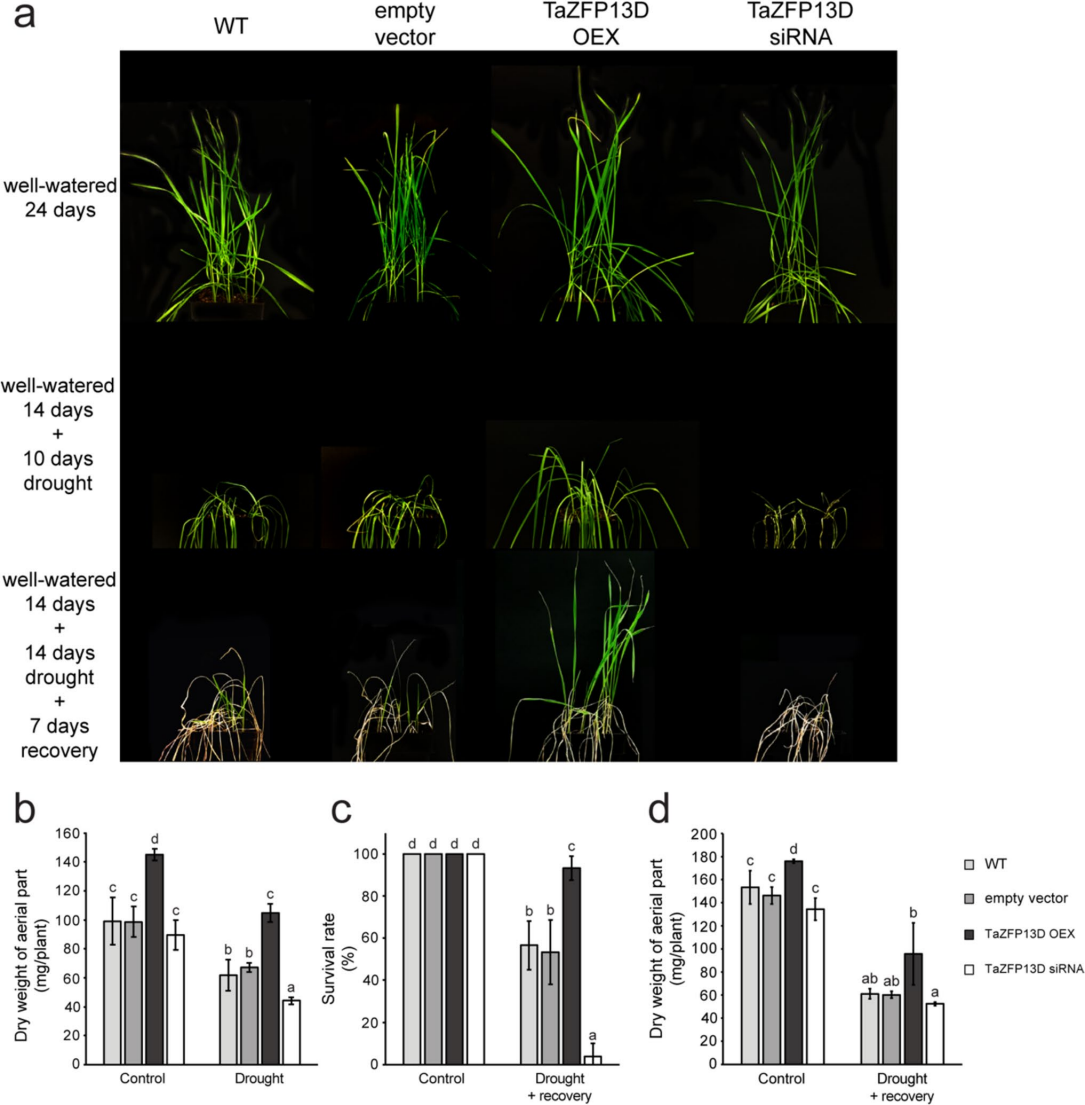
TaZFP13D positively regulates growth and drought tolerance in the wheat cultivar Atlas66*. TaZFP13D* expression was modulated in the wheat cultivar Atlas66 using the BSMV expression system. Plants were grown and treated under the conditions indicated on the figure. (**a)** Representative photographs of the plants. (**b)** Biomass production was assessed by quantifying the dry weight of the plants’ aerial parts. (**c)** For re-growth experiments, the survival rate was determined as the percentage of plants able to regrow after the recovery period. (**d)** Biomass production was determined after the recovery period. Data are means +/− S.D. of at least three biological replicates of groups of 10 plants, and statistical analysis was performed by a one-way ANOVA followed by a Tuckey’s test (*P* < 0.05). Different letters indicate a statistical difference.

We previously showed that *TaZFP13D* plants overexpressing this gene showed less wilting after a short term water stress (water withheld for 7 days) (Bouard and Houde 2022). To evaluate TaZFP13D’s capacity to minimize severe drought stress effects, plants were exposed to longer dehydration treatments (water withheld up to 14 days). After withholding water for 10 days, the drought-induced wilting phenotype was much less pronounced in OEX plants compared to WT and empty vector plants (Fig. 3a). On the opposite, leaf wilting was more severe in siRNA plants compared to WT and empty vector plants (Fig. 3a). The apparent increase in drought tolerance for OEX plants might be related in part to an overall effect of TaZFP13D on growth. Well-watered 24-day-old OEX plants looked bigger compared to the other types of plants (Fig. 3a), therefore biomass was quantitated as dry weight. Well-watered 24-day-old WT (99.1 mg/plant), empty vector (98.7 mg/plant) and siRNA plants (89.4 mg/plant) showed similar biomass, while OEX plants biomass production was significantly higher (145.2 mg/plant) (Fig. 3b). For all types of plants, withholding water for 10 days reduced biomass production but the decrease was less in OEX plants (104.7 mg/plant) compared to WT (61.8 mg/plant) and empty vector plants (67.1 mg/plant). On the opposite, biomass produced by drought-treated siRNA plants (44.1 mg/plant) was significantly decreased (Fig. 3b).

The plants’ capacity to recover after a severe drought stress was also examined. Approximately half of the control plants (57% and 53% for WT and empty vector plants, respectively) survived after 14 days without water and 7 days of recovery (Fig. 3c). By contrast, the survival rate of OEX plants was almost unaffected (93% survival) by this stress, while a dramatic reduction (4% survival) was observed for siRNA plants (Fig. 3c). Additionally, OEX plants showed a remarkably superior re-growth capacity compared to the other types of plants (Fig. 3a). Consequently, 35-day-old OEX plants also showed a significantly higher dry biomass after drought recovery compared to the other types of plants (Fig. 3d).

### TaZFP13D reduces drought-induced ROS

**Fig. 4:**
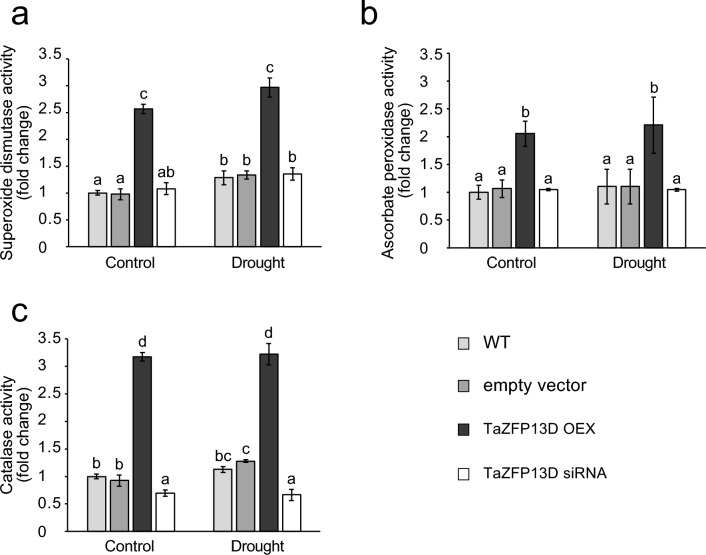
*TaZFP13D* overexpression improves antioxidant enzymes activity in the wheat cultivar Atlas66. **(a)** Superoxide dismutase (SOD), (**b**) ascorbate peroxidase (APX) and (**c**) catalase (CAT) enzymatic activities were determined in soluble extracts from second leaves of wild-type (WT), empty vector, *TaZFP13D* OEX and siRNA plants grown under well-watered conditions for 21 days (Control), or for 14 days then drought-treated for 7 days (Drought). Data are means +/− S.D. of three biological replicates, and statistical analysis was performed by one-way ANOVA followed by a Tuckey’s test (*P* < 0.05). Different letters indicate a statistical difference.

**Fig. 5:**
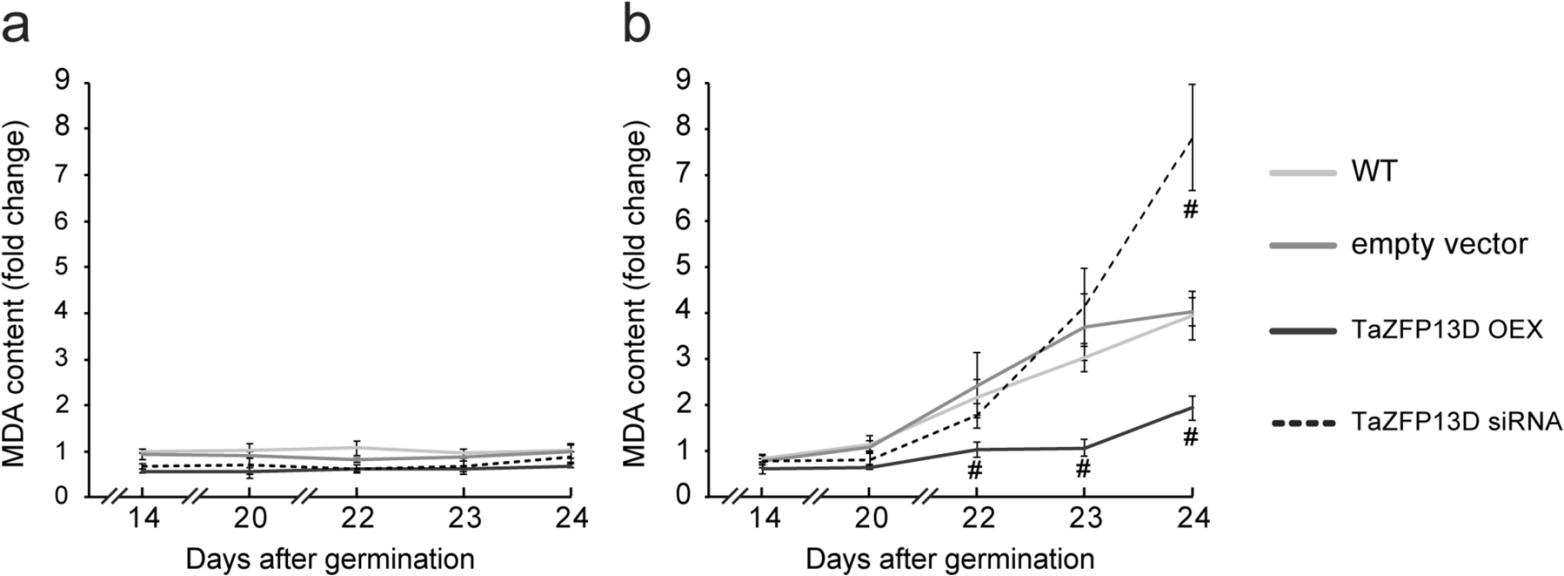
TaZFP13D overexpression reduces drought-induced oxidative damage to lipids in the wheat cultivar Atlas66. Malondialdehyde (MDA) content was quantified from leaf tissue samples harvested from wheat seedlings at different times over a 24-day period. Plants were grown under well-watered conditions for 24 days (**a**) or for 14 days under well-watered conditions followed by a drought treatment performed by withholding water for up to 10 days (**b**). MDA content (mmol per gram of dry weight tissue) was determined by TBARS. Data are means +/− S.D. of three biological replicates and statistical analysis was performed by one-way ANOVA followed by a Tuckey’s test (P < 0.05). The “#” symbol indicates a statistical difference compared to WT and empty vector plants.

The activity of antioxidant enzymes was measured in seedlings of the various wheat plants and conditions. SOD (Fig. 4a), APX (Fig. 4b) and CAT (Fig. 4c) activity was 2.5-3-fold higher in OEX plants compared to the other types of plants, under well-watered growth condition (control) and after 7 days of drought treatment (drought). Oxidative damage includes, among others, lipid peroxidation. This can be monitored by quantifying malondialdehyde (MDA), a by-product of lipid peroxidation. When grown under well-watered conditions, all types of plants showed a similar and stable MDA accumulation for all tested time points (Fig. 5a). Withholding water for 10-days significantly increased MDA accumulation in leaves by 4-fold in WT and empty vector plants (Fig. 5b). More importantly, the MDA level in OEX plants after 10 days of drought treatment was half the level observed in WT and empty vector plants, while siRNA plants had twice as much MDA compared to the controls (Fig. 5b). These results confirm that TaZFP13D is associated with a higher protection of plants against drought-induced oxidative stress.

### Transcriptome analyses

**Fig. 6:**
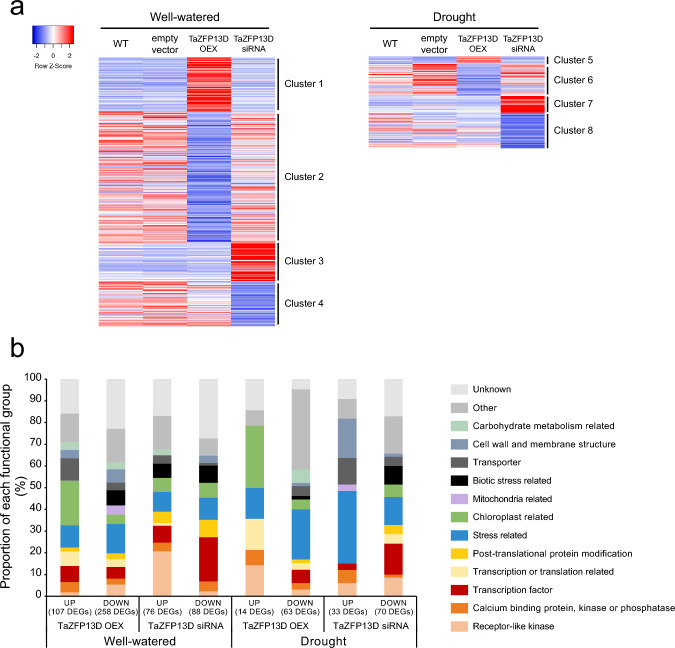
Differentially expressed genes in wheat lines that over- or underexpress *TaZFP13D*. The four types of plants (Wild-type, empty vector, *TaZFP13D* OEX and siRNA) were grown under well-watered conditions for 21 days (Well-watered) or for 14 days before withholding water for 7 days (Drought). RNA was extracted from leaf tissue samples and twelve mRNA libraries were generated and sequenced to identify differentially expressed genes (DEGs) in OEX and siRNA plants using the DESeq2 software. DEGs showing a Log 2-fold change ≥ 1.5 or ≤ − 1.5 with an adjusted p-value (FDR) < 0.001 compared to all other types of plants, cultivated in the same growth conditions were selected. (**a**) Heatmaps showing the expression pattern of each DEGs cluster. (**b**) Classification of the selected DEGs based on their functional annotation. UP: up-regulated; DOWN: down-regulated.

To better understand the function of TaZFP13D, we used RNA-Seq to identify DEGs in OEX and siRNA plants grown under well-watered (control) or drought conditions. The identified up- or down-regulated genes were grouped into 8 clusters and a unique gene number (#) was given to each DEG to facilitate referencing hereafter (Online Resource 2, Fig. 6a). Our selection of DEGs revealed that 442 (clusters 1, 2, 5 and 6) and 267 (clusters 3, 4, 7 and 8) non-redundant genes were differentially expressed in OEX and siRNA plants, respectively (Online Resource 2). The comparison of DEGs identified in OEX plants grown under well-watered (clusters 1 and 2) and drought conditions (clusters 5 and 6) reveals that only 1.8% of these are common, suggesting that TaZFP13D regulates different target genes under normal growth conditions and during drought stress. The expression level of these DEGs is illustrated on heatmaps, highlighting the expression pattern of each cluster (Fig. 6a). Based on annotation, DEGs were grouped into functional categories (Online Resource 2, Fig. 6b). This classification revealed that TaZFP13D mostly regulates genes involved in signal perception, signal transduction, transcriptional regulation, stress response and chloroplast regulation (Fig. 6b).

To identify the molecular and cellular functions responsible for the enhanced growth performances and drought tolerance conferred by TaZFP13D, we focused our analysis on DEGs identified in OEX plants (clusters 1, 2, 5 and 6) since it is the only type of plants exhibiting a significant improvement of these traits (Fig. 3). Analysis revealed that many receptor-like kinases (RLKs) were down-regulated by *TaZFP13D* OEX in well-watered conditions (Fig. 6b and Online Resource 2, genes #108-121). Among these differentially expressed RLKs, known to be important in balancing plant growth and stress responses, we identified several members of the leucine-rich repeat RLKs (LRR-RLK), cysteine-rich kinases (CRK), lectin RLKs (LecRLK), and wall-associated kinase (WAK) sub-families. In addition, we identified several up- or down-regulated DEGs involved in signal transduction of stress responses (Fig. 6b and genes #3-7, #122-130), which are mediated by post-translational protein modifications like phosphorylation and secondary messengers such as calcium (Ca^2+^), inositol-1,4,5-trisphosphate (IP3) or H_2_O_2_ (Zhang et al. 2022). For example, some protein kinases belonging to the calcium-dependent protein kinase (CDPK) and the mitogen-activated protein kinase kinase kinase (MAP3K) families were differentially expressed (genes #6 and #122 respectively), concomitantly with several phosphatases like inositol polyphosphate 5-phosphatase (5PTase) and protein phosphatase 2C (PP2C) (genes #3 and #123 respectively). Moreover, three genes related to Ca^2+^ signaling were down-regulated in OEX plants (genes #126, #129-130). These DEGs are known to regulate stress responses and may modulate response sensitivity (Chen et al. 2021; Perera et al. 2008).

Several up- and down-regulated transcription factors were identified in OEX plants (Fig. 6b, genes #8-15 and #131-142, respectively). Among them, we identified DEGs belonging to families like dehydration-responsive element-binding protein (DREB), basic leucine zipper (bZIP), nuclear factor Y (NF-Y), myeloblastosis oncogene (MYB), abscisic acid-, stress- and ripening-induced (ASR), and genes encoding transcription factors containing the conserved WRKYGQK motif (WRKY). These transcription factor families are known to be an important part of stress response regulatory networks and often contain members that act as positive or negative regulators of stress tolerance (Hu et al. 2022).

We identified several DEGs related to biochemical or metabolic stress responses in well-watered OEX plants (Fig. 6b, #16-26, #143-177). Many of these genes encode proteins associated with oxidative response and redox homeostasis like lipoxygenase, peroxidase, oxalate oxidase, cystathionine beta synthase (CBS) domain-containing protein, respiratory burst oxidase homolog protein B-like (RBOHB), thioredoxins (TRXs), aldehyde dehydrogenase, glutathione S-transferase and heat shock proteins (HSP). These results indicate that oxidative stress responses are modulated in OEX plants under normal growth conditions. More importantly, classification of DEGs into functional categories revealed that well-watered OEX plants up-regulated many genes related to chloroplast functions (Fig. 6b, genes #27-48). Many of these genes are involved in the production of key PETC protein complexes such as the NAD(P)H dehydrogenase (NDH) complex, which is known to have important functions for photosynthesis efficiency, growth, ROS regulation and adaptation to stress (Yamori and Shikanai 2016).

As already mentioned, DEGs in OEX and siRNA plants were retained if their expression level was above the selected cut-off compared to all other types of plants cultivated under the same growth conditions. While drought treatment significantly increased *TaZFP13D* expression in control plants (WT and empty vector) by 8-fold, drought-treated OEX plants only showed a 1.5-fold increase compared to drought-treated control plants (Fig. 2c). Thus, it is not surprising that the number of DEGs identified in drought-treated OEX plants is reduced (77 DEGs, Online Resource 2) compared to well-watered ones (365 DEGS, Online Resource 2). Among these 77 DEGs, we identified up-regulated genes encoding Rubisco small chain (RBCS) and Rubisco Activase (RCA) B (#533-535). In addition, several DEGs related to the drought response were up-regulated. These include genes involved in important mechanisms required for cellular adaptation to drought like stomatal closure (#532) or cell wall loosening (#538). Concomitantly, we identified several genes encoding HSPs (#171, #197, #198) that were down-regulated in both well-watered and drought-treated OEX plants. These genes are known to be up-regulated in response to ROS, suggesting that levels of these molecular species are reduced in OEX plants. These results support that drought stress intensity was mitigated in OEX plants, which is in accordance with the plant’s drought stress tolerance phenotype (Fig. 3).

**Fig. 7:**
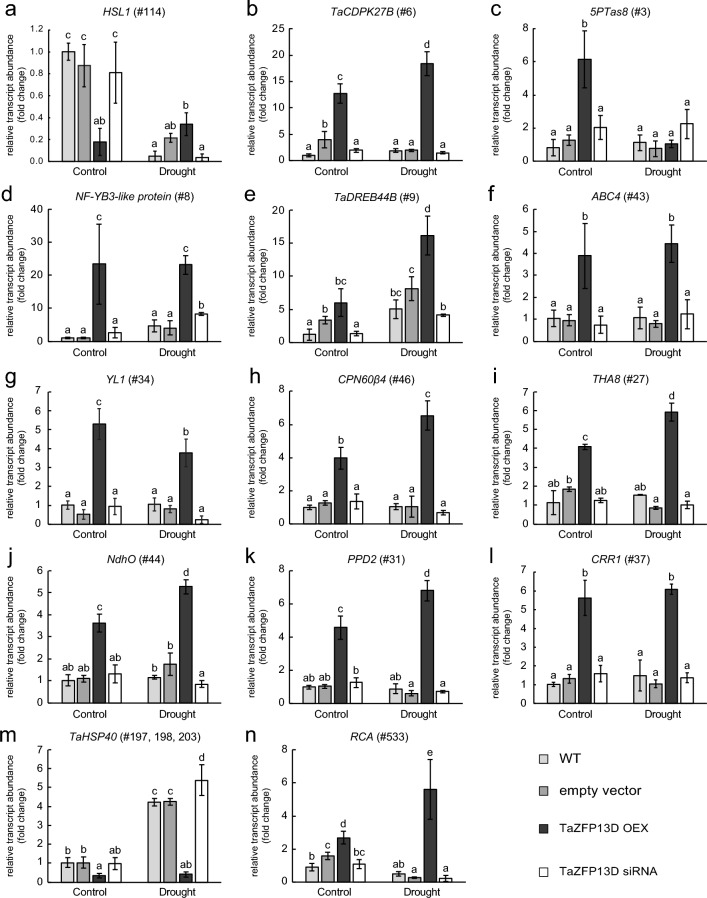
qRT-PCR validation of selected differentially expressed genes identified by RNA-Seq. To confirm the expression data obtained from the RNA-Seq analysis, 3 new biological replicates were generated for each type of plants: wild-type (WT), empty vector, *TaZFP13D* OEX and siRNA. Plants were grown and treated as described in Fig. 2 and the second leaves were harvested. Total RNA was extracted, reverse-transcribed and used for qRT-PCR analysis of selected genes. (**a**)_ HSL1: HAESA-Like 1. (**b**) CDPK: calcium-dependent protein kinase. (**c**) 5PTase8: inositol polyphosphate 5-phosphatase 8. (**d**) NF-YB: nuclear factor Y subunit B. (**e**) DREB: dehydration-responsive element-binding protein. (**f**) ABC4: DHNA-phytyltransferase. (**g**) YL1: Yellow Leaf protein 1. (**h**) CPN60: chaperonin 60 subunit b4. (**i**) THA8: thylakoid assembly protein 8. (**j**) NdhO: NAD(P)H dehydrogenase subunit O. (**k**) PPD2: PsbP domain-containing protein 2. (**l** CRR1: chlororespiratory reduction protein 1. (**m**) HSP: heat shock protein. (**n**) RCA: Rubisco Activase B. The high similarity between the homoeologous genes in (**d**), (**j**)**, **(**k**) and (**n**) did not allow for the design of specific primer pairs, therefore sub-genome copies A, B and D were measured concomitantly. The gene numbers indicated in each panel (#) refer to the gene numbers in Online Resource 2. See Online Resource 1 for primers and targets. Data are means +/− S.D. of three biological replicates, and statistical analysis was performed by one-way ANOVA followed by a Tuckey’s test (P < 0.05). Different letters indicate a statistical difference.

To confirm the expression patterns observed in the RNA-Seq data, we have selected key DEGs based on their functions (see discussion) and validated their expression by qRT-PCR using three new biological replicates for each plant type and condition (Fig. 7). This analysis confirmed the expression patterns for all tested genes (Fig. 7). Supporting this observation, the evaluation of the correlation between expression levels obtained from RNA-Seq and qRT-PCR data (Online Resource 3) showed a good Pearson correlation coefficient (*r* = 0.79). However, slight variations in relative expression level were detected between RNA-Seq and qRT-PCR data, which impact the strength of the model (R^2^ = 0.63).

## Discussion

### TaZFP13D improves growth and drought tolerance

In this work, we aimed to characterize the function of the TaZFP13D transcription factor using plants that over- (OEX) and underexpress (siRNA) the corresponding gene. *TaZFP13D* OEX plants showed increased growth under well-watered growth conditions and drought stress compared to WT and empty vector plants, suggesting a better carbon assimilation capacity under these growth conditions. Our results demonstrate that TaZFP13D improves drought tolerance and strongly increases survival rates and recovery after a severe drought treatment (Fig. 3). As expected, siRNA plants were more sensitive to drought, indicating that TaZFP13D function is essential for drought tolerance and that the decrease in transcript level cannot be significantly compensated by other wheat genes (Fig. 3).

**Fig. 8:**
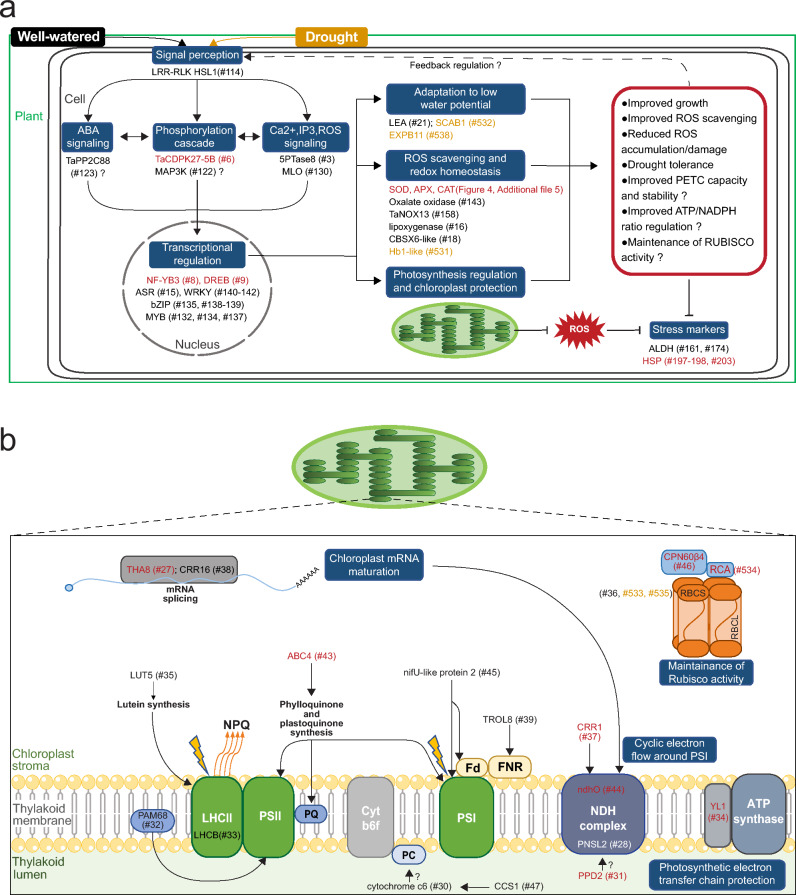
Model summarizing putative TaZFP13D-dependent molecular, biochemical and physiological events. Illustration of major changes in gene expression regulated by TaZFP13D (**a**). A more detailed illustration is presented for genes associated with chloroplast structure or function (**b**). The symbol (#) refers to the gene numbers in Online Resource 2. Genes illustrated in black and orange were respectively identified under well-watered and drought conditions, while genes illustrated in red are differentially expressed under both conditions. Black arrows indicate known functions, based on previous characterization of similar genes. LRR-RLK HSL1: leucine-rich repeat receptor-like kinase HAESA-Like 1, ABA: abscisic acid, PP2C: protein phosphatase 2C, CDPK: calcium-dependent protein kinase, MAP3K: mitogen-activated protein kinase kinase kinase, Ca^2+^: calcium, IP3: Inositol-1,4,5-trisphosphate, ROS: reactive oxygen species, 5PTase: inositol polyphosphate 5-phosphatase, MLO: Mildew Resistance Locus-like protein 1, NF-YB: nuclear factor Y subunit B3-like protein, DREB: dehydration-responsive element-binding protein, ASR: abscisic acid-, stress- and ripening-induced, WRKY: transcription factors containing the conserved WRKYGQK motif, bZIP: basic leucine zipper, MYB: myeloblastosis oncogene, LEA: late embryogenesis abundant protein, SCAB1: stomatal closure-related actin-binding protein 1, EXPB11: β-expansin11, SOD: superoxide dismutase, APX: ascorbate peroxidase, CAT: catalase, NOX: NADPH-oxidase respiratory burst oxidase homolog protein B-like, CBSX: cystathionine beta synthase domain-containing protein, Hb1-like: non-symbiotic hemoglobin 1-like, PETC: photosynthetic electron transport chain, NADPH: reduced nicotinamide adenine dinucleotide phosphate, ALDH: aldehyde dehydrogenase, HSP: heat shock proteins, THA8: thylakoid assembly protein 8, CRR: chlororespiratory reduction protein, RNP: ribonucleoprotein, WTF1: What’s this factor 1, PPR: pentatricopeptide repeat protein, LUT: lutein, PAM68: photosynthesis affected mutant protein 68, LHC: Light-Harvesting Complex, ABC4: 2-carboxy-1,4-naphthoquinone phytyltransferase, PS: photosystem, PQ: plastoquinone, Cyt b6f: Cytochrome b6f, PC: plastocyanin, CCS1: Cytochrome c biogenesis protein 1, TROL8: thylakoid rhodanese-like protein 8, Fd: ferredoxin, FNR: ferredoxin:NADP^+^ oxidoreductase, NDH: NAD(P)H dehydrogenase complex, PNSL2: photosynthetic NDH subunit of lumenal location 2, PPD2: PsbP domain-containing protein 2, YL1: Yellow Leaf protein 1, Rubisco: ribulose-1,5-bisphosphate carboxylase/oxygenase, RBCS: Rubisco small chain, RBCL: Rubisco large chain, RCA: Rubisco Activase B, CPN60: chaperonin 60 subunit b4.

The transcriptome analysis performed in this study allowed the identification of many genes regulated by TaZFP13D (Online Resource 2, Fig. 6). Comparison of the DEG clusters revealed that TaZFP13D regulates almost completely different gene subsets under well-watered conditions and during drought stress. This shift in TaZFP13D activity could depend on the interaction partners present under either well-watered or drought conditions. Another possibility is that post-translational modifications (PTMs) could occur in one of the growth conditions but not in the other. One of these possible PTMs that might occur during drought is phosphorylation, since we identified several putative phosphorylation sites on TaZFP13D (Bouard and Houde 2022). Considering this difference in genes regulated by TaZFP13D between growth conditions, transcriptome modifications under well-watered and drought conditions will be discussed separately. Based on functional annotation, we showed that many DEGs are involved in signal perception, signal transduction, regulation of gene expression, stress response or chloroplast regulation (Fig. 6b). These processes are important for the plant’s responses to stress and may play a role in stress tolerance (Zhang et al. 2022). However, many of these DEGs have not yet been characterized in wheat or in a drought context even though many of them belong to stress-related gene families. For this reason, we focused on DEGs (or their close relatives) which have been functionally characterized by gene overexpression or knock-down/knock-out experiments in wheat or other model species to better understand TaZFP13D function in the growth improvement and drought tolerance observed in OEX plants. A model illustrating relevant changes in gene expression mediated directly or indirectly by TaZFP13D is proposed to support the discussion (Fig. 8).

### Modulation of gene expression in OEX plants under well-watered conditions

#### Signal perception and transduction

Under well-watered growth conditions, many RLKs belonging to LRR-RLK, CRK, LecRLK, and WAK sub-families are differentially expressed in OEX plants (Fig. 6b and Online Resource 2). There is increasing evidence that these RLK sub-families are important for signal perception and play crucial roles in balancing growth and stress responses (with positive and negative regulators), including oxidative and drought stresses (Ye et al. 2017; Zhu et al. 2023). However, the RLK family is extremely vast, and characterization of specific RLK function is difficult due to sequence similarity between members, interactions with co-receptors, and cross-talks between environmental adaptation responses. Thus, many RLKs have not been characterized in wheat, including most of the differentially expressed RLKs identified in this study. Among the characterized RLKs, we identified a gene coding for HAESA-Like 1 (HSL1) receptor-like protein kinase (#114) that is down-regulated in well-watered OEX plants (Online Resource 2, Figs. 7a and 8a). Previous work demonstrated that HSL1 is important for stomatal development in Arabidopsis (Qian et al. 2018). Reducing stomata density, a common response to drought, increases dehydration tolerance since transpiration rate and soil drying are decreased (Dunn et al. 2019). Interestingly, these authors showed that there is no loss in plant productivity when stomatal density is moderately reduced (Dunn et al. 2019). This suggests that TaZFP13D could be involved in decreasing stomatal density under well-watered conditions, and may participate in improving water use efficiency under well-watered conditions. This could also help OEX plants to better tolerate drought conditions.

A transient increase in cytosolic Ca^2+^ is an early step in the sensing of many stresses, which is decoded by calcium-binding proteins (Allan et al. 2022; Xu et al. 2022). We identified a down-regulated gene coding for a Mildew Resistance Locus (MLO)-like protein 1 (#130) in OEX plants, while the same gene (#130) and another gene coding for a similar protein (#383) are up-regulated in siRNA plants (Online Resource 2). The MLO gene family members encode stress-responsive transmembrane calmodulin-binding proteins (Pépin et al. 2021; Piffanelli et al. 2002), and a previous study showed that pepper CaMLO2 is a negative regulator of ABA sensitivity and drought tolerance (Lim and Lee 2014). This suggests that TaZFP13D negatively regulates these MLO-like protein 1 and may thus increase ABA-sensitivity in OEX plants (Fig. 8a). Additionally, we identified an up-regulated CDPK (#6) in OEX plants (Online Resource 2, Fig. 7b). This gene family is involved in Ca^2+^-dependent stress sensing, and plays a key role in abiotic stress tolerance (Atif et al. 2019). For example, characterization of TaCDPK34 in wheat highlighted its positive role in drought tolerance (Li et al. 2020). Another study demonstrated that TaCDPK27B (#6) is important for salt tolerance and stress-induced ROS protection in wheat (Yue et al. 2022). Up-regulation of this gene in OEX plants may contribute to the increased ROS scavenging enzyme activities (Fig. 4).

A gene encoding a 5PTase8 (#3) is up-regulated in well-watered OEX plants (Online Resource 2 and Fig. 7c). IP3 is another well-known secondary messenger involved in Ca^2+^ and ABA signaling, as it can be hydrolyzed by 5PTases (Jia et al. 2019). Several 5PTases were associated with abiotic stress tolerance in Arabidopsis, including oxidative stress and drought. For example, overexpression of a mammalian type 1 5PTase in Arabidopsis alters Ca^2+^ and ABA signaling, which improves drought tolerance, stomatal response to ABA, and DREB gene expression (Perera et al. 2008). Similarly, overexpression of 5PTase in tomato improves biomass production under well-watered growth conditions, while water loss is decreased upon drought treatment (Khodakovskaya et al. 2010). Up-regulation of a 5PTase8 (#3) in OEX plants suggests that *TaZFP13D* overexpression may improve IP3 hydrolysis and contribute to improve biomass production, ROS scavenging and ABA sensitivity in this type of plant (Figs. 3, 4 and 8a).

#### Transcriptional reprogramming

It is well known that drought stress response and tolerance are mediated by a regulatory network controlled by many transcription factor families like DREB, bZIP, NF-Y, MYB, ASR or WRKY (Hrmova and Hussain 2021; Hu et al. 2022). Additionally, drought response is tightly associated with hormonal growth regulation, which mainly involves ABA and cross-talks with other phytohormones like salicylic acid, brassinosteroid, ethylene or jasmonate (Iqbal et al. 2022; Muhammad Aslam et al. 2022). Consistently, most of the differentially expressed transcription factors identified belong to these families (Online Resource 2 and Fig. 8a) and may be important to properly regulate many effectors involved in stress response. However, the function of specific members in wheat remains poorly understood. Nevertheless, well-watered OEX plants have an up-regulated gene coding for a nuclear factor Y subunit B (NF-YB)-3-like protein (#8) (Fig. 7d), which may contribute to the observed phenotype since overexpression of closely related NF-YB proteins improves drought tolerance, ROS scavenging enzymes activities, photosynthetic rate, and ABA-mediated closure in Arabidopsis, tobacco, soybean, and maize (Nelson et al. 2007; Sun et al. 2022; Zhang et al. 2015; Zhao et al. 2022). In addition, OEX plants also up-regulate TaDREB44B (#9) (Online Resource 2, Figs. 7e and 8a), which belongs to the well-known DREB/CBF family involved in dehydration response and drought tolerance (Niu et al. 2020). The overexpression of this gene in well-watered plants may be related to the greater ABA sensitivity suggested above and may contribute to improve the drought response.

#### Photosynthesis regulation and chloroplast protection

Considering the crucial functions of chloroplasts in growth regulation and ROS production, genes genes encoding proteins involved in chloroplast functions particularly caught our attention since they constitute the largest group of up-regulated genes in *TaZFP13D* OEX plants (Fig. 6b).

Among these up-regulated genes, we identified an upregulated Chlorophyll a/b binding protein of light-harvesting complex (LHC) II type 1-like (#33) in well-watered OEX plants (Online Resource 2, Fig. 8b). Chlorophyll a/b binding proteins (also known as CABs or LHCBs) are pigment-binding proteins that are part of the photosystem antennas and play a crucial role in harvesting light for photosynthesis and redirecting excess energy as heat (non-photochemical quenching, NPQ), depending on cell demand (Mascoli et al. 2019). Previous work demonstrated that LHCB overexpression in Arabidopsis or tobacco increases biomass production in normal growth conditions and increases NPQ under high light treatment (Aghdasi et al. 2012; Labate et al. 2004). Moreover, it has been shown that drought tolerant wheat cultivars accumulate more LHCB at the transcriptional and protein levels in both well-watered growth conditions and upon drought treatment (Liu et al. 2006). Similarly, OEX plants up-regulate CYP97A3 (#35), also known as Lutein Deficient 5 protein (LUT5) in Arabidopsis, involved in lutein synthesis (Kim and DellaPenna 2006). When bound to LHCBs, lutein stabilizes the antenna protein structure and participates in light harvesting (Jahns and Holzwarth 2012). In addition, lutein has been reported to play an important role in NPQ (Li et al. 2009; Mascoli et al. 2019) and photoprotection (Dall’Osto et al. 2006), suggesting that TaZFP13D improves NPQ, photosystems protection, and thus photosynthetic efficiency through improved Chlorophyll a/b binding protein and lutein synthesis (Fig. 8b). We also identified an up-regulated gene coding for a photosynthesis affected mutant (PAM) 68 protein (#32). Previous work in Arabidopsis showed that PAM68 mutation severely impacts growth, PSII assembly and activity (Armbruster et al. 2010). More precisely, PAM68 is involved in D1 protein biogenesis and PSII core protein stability (CP43 and CP47 accumulation) (Armbruster et al. 2010). It is well known that D1 protein is the most susceptible site of oxidative damages within the PETC, even under normal growth conditions, due to the water oxidation property of PSII (Kale et al. 2017; Li et al. 2018). In addition, plants constantly replace damaged D1 proteins, which involves substantial synthesis of this protein. However, this process is inhibited by ROS overproduction, which leads to photo-inhibition (Gururani et al. 2015). This suggests that *TaZFP13D* overexpression contributes to PSII protection and improvement of photosynthesis by increasing D1 biosynthesis through PAM 68 up-regulation.

Overexpression of *TaZFP13D* up-regulates the gene encoding 2-carboxy-1,4-naphthoquinone phytyltransferase (ABC4) (Online Resource 2 and Fig. 7f), also known as DHNA-phytyltransferase (#43). This enzyme is involved in phylloquinone and plastoquinone synthesis, two quinones involved in electron transfer in the PSI and PSII photosystems, respectively (Guergova-Kuras et al. 2001; Van Eerden et al. 2017). Mutation of this gene in Arabidopsis drastically reduces phylloquinone and plastoquinone levels, which destabilizes PSI and PSII core proteins, impairs electron transfer from PSII to PSI, alters thylakoid structure and confers a pale-green phenotype (Shimada et al. 2005). Under well-watered growth conditions, a higher ABC4 activity in *TaZFP13D* OEX plants may provide better electron flow capacity and carbon assimilation, which contribute to improve biomass production and avoid PETC over-reduction (Fig. 3).

Overexpression of *TaZFP13D* up-regulates a gene coding for a chloroplastic nifU-like protein 2 (#45). In Arabidopsis, the nifU-like protein 2 is important for photosynthetic electron transport and growth since it contributes to the biosynthesis of the iron-sulfur clusters of PSI and ferredoxin (Touraine et al. 2004; Yabe et al. 2004). These observations suggest that TaZFP13D may improve PSI and ferredoxin assembly or abundance, preventing PETC over-reduction by increasing its capacity (Fig. 8b). Further supporting the important role of TaZFP13D in PETC regulation, we identified an up-regulated gene coding for thylakoid rhodanese-like protein 8 (TROL8) (#39) in OEX plants. Previous work in Arabidopsis demonstrated that TROL proteins are important for tethering of ferredoxin:NADP^+^ oxidoreductase (FNR) to the thylakoid membrane and to sustain linear electron flow efficiency (Jurić et al. 2009). Such association, which is necessary to maintain photosynthetic activity, might be improved in OEX plants.

In the same line of evidence, a gene coding for a protein Yellow Leaf 1 (YL1) is up-regulated in well-watered OEX plants (Online Resource 2, #34, Figs. 7g and 8b). Previous work performed in rice showed that YL1 is important for chloroplastic ATP synthase biogenesis and activity (Chen et al. 2016). This suggests that assembly of chloroplastic ATP synthase may be more efficient in OEX plants to enhance photosynthesis and carbon assimilation.

Further supporting the positive effect of TaZFP13D on photosynthesis efficiency, we identified an up-regulated gene encoding chaperonin (CPN) 60β4 (Online Resource 2, #46, Figs. 7h and 8b), which is known to be important for Rubisco and Rubisco activase (RCA) structure and function (Salvucci 2008; Vitlin Gruber and Feiz 2018).

Two up-regulated genes in OEX plants (#38 and #27) encode respectively for a chlororespiratory reduction protein (CRR) 16 and a thylakoid asssembly (THA) protein 8 (Fig. 7i). These ribonucleoproteins (RNPs) are involved in chloroplastic mRNA group II intron splicing (Khrouchtchova et al. 2012; Yamamoto et al. 2020). Previous work in Arabidopsis demonstrated that CRR16 is important for efficient ndhA splicing and function, which is required for chloroplastic NDH complex activity and stability (Yamamoto et al. 2020). Moreover, functional characterization of THA8 in maize showed its important function in protein translocation across the thylakoid membrane, which is crucial to maintain high thylakoid membrane protein complex abundance (Khrouchtchova et al. 2012). These observations suggest that TaZFP13D improves thylakoid protein complexes abundance through chloroplastic mRNA maturation, including the NDH complex, well-known to be involved in PETC protection by mediating cyclic electron flow (CEF) around PSI (Fig. 8b) (Yamori and Shikanai 2016). Regulation of chloroplastic mRNA maturation improving NDH complex abundance was reported previously in rice, during the characterization of the RNP OsCRP1 (Bang et al. 2021). OsCRP1 was shown to bind and up-regulate NDH complex related transcripts by approximately 2.5-fold, resulting in enhanced CEF mediated by the NDH complex and ATP production (Bang et al. 2021). Accordingly, we identified two up-regulated genes in OEX plants encoding important proteins of the chloroplastic NDH complex: NdhO (#44; Fig. 6j) and photosynthetic NDH subunit of lumenal location 2 (PNSL2 or PQL2; #28) (Ma et al. 2021). Previous work showed that NdhO and PNSL2 mutations in Arabidopsis completely abolished NDH complex activity and severely impaired complex formation (Rumeau et al. 2005; Suorsa et al. 2010). In addition, OEX plants up-regulate a gene coding for a psbpomain-containing protein 2 (PPD2) (#31; Fig. 7k), which is co-expressed with many genes related to the NDH complex in Arabidopsis, including PNSL2/PQL2 (Ifuku et al. 2010). Moreover, the CRR1 gene (#37) is also up-regulated in well-watered OEX plants (Online Resource 2, Figs. 7l and 8b). In Arabidopsis, CRR1 is involved in NDH complex assembly and mutation of this gene impairs NDH complex activity and accumulation (Shikanai 2016; Shimizu and Shikanai 2007). These DEGs related to NDH complex suggests that *TaZFP13D* overexpression improves NDH mediated CEF (Fig. 8b). This alternative route for electron within PETC increases the ΔpH across the thylakoid membrane and promotes ATP production, an important process to regulate ATP/NADPH ratio and photosynthetic efficiency (Kramer and Evans 2010; Ma et al. 2021; Munekage et al. 2004; Shikanai 2016; Shikanai and Yamamoto 2017; Yamori and Shikanai 2016). In addition, thylakoid lumen acidification generated by CEF induces NPQ, which protects the PETC from over-reduction and photo-damage by eliminating excess light energy through thermal dissipation (Shikanai and Yamamoto 2017; Yamori and Shikanai 2016).

Taken together these results suggest that *TaZFP13D* overexpression improves abundance, assembly, stability, and quality control of many key PETC effectors (Fig. 8b). The DEGs may improve PETC capacity, photosystems protection and photosynthesis efficiency under well-watered growth conditions, contributing to the increase in biomass observed in OEX plants (Fig. 3). Detailed photosynthetic measurements will be needed to confirm the effect of TaZFP13D on PETC.

#### Oxidative stress protection and redox homeostasis

In a previous work, we demonstrated that *TaZFP13D* overexpression improves SOD, APX and CAT activity in the wheat lines NIL103 and NIL106, under well-watered conditions or drought stress, and that drought-treated plants of the wheat cultivar Atlas66 accumulate less H_2_O_2_ and superoxide anions (Bouard and Houde 2022). In the present study, we confirmed that TaZFP13D increases the activity of key antioxidant enzymes and reduces oxidative damage to lipids in the wheat cultivar Atlas66 (Figs. 4 and 5). Surprisingly, we did not identify any DEGs coding for SOD, APX and CAT in OEX plants using a 1.5 Log 2-fold change cut-off and an adjusted p-value < 0.001 (Online Resource 2). A closer analysis revealed that many of these genes are up-regulated, although their Log 2-fold change or adjusted p-value were below the threshold used to select TaZFP13D DEGs (Online Resource 6). The qRT-PCR results confirmed that SOD, APX and CAT expression is significantly higher in OEX plants under both well-watered and drought conditions (Online Resource 6), which supports the increase in enzyme activity (Fig. 4). However, siRNA plants showed similar SOD, APX and CAT activity as control plants (WT and Empty vector) but accumulated approximately twice more MDA after 10 days of drought (Fig. 5b). This suggests that other ROS scavenging systems may be down-regulated in siRNA plants or some ROS producing mechanisms are more active in this type of plants. The possibility that these metabolic adjustments could be impaired in siRNA plants and the lower expression of TaZFP13D in these plants are in agreement with a possible role of TaZFP13D in the improvement of photosynthesis and chloroplast protection against drought-induced ROS damage.

In addition to the enhanced activity of ROS scavenging enzymes, transcriptome analysis revealed that well-watered OEX plants strongly down-regulate some genes encoding ROS generating proteins like oxalate oxidase GF-2.8-like (#143) or the RBOHB TaNOX13 (#158). Concomitantly, OEX plants have an up-regulated gene coding for a putative linoleate 9 S-lipoxygenase 3 (#16), which may contribute to their stress-tolerant phenotype (Fig. 8a) since lipoxygenases are known to be important for development, hormonal responses and drought tolerance (Lim et al. 2015; Singh et al. 2022; Xing et al. 2020). The balance between ROS production and scavenging is important for redox homeostasis, stress perception and stress response (Foyer and Noctor 2005). Well-watered OEX plants up-regulate a CBSX6-like protein (#18) (Online Resource 2, Fig. 8a). Previous studies using transgenic plants demonstrated that CBSX proteins can activate TRXs, improve plant growth, reduce H_2_O_2_ content in leaves and enhance abiotic stress tolerance, including drought (Murai et al. 2021; Serrato et al. 2013; Singh et al. 2012; Yoo et al. 2011). This result suggests that TaZFP13D may improve plant growth and drought tolerance through CSBX and TRXs, which are key sensors of the cellular redox state and important regulators of many physiological processes, including photosynthesis and abiotic stress responses (Gelhaye et al. 2005).

Taken together, these results show that *TaZFP13D* overexpression under well-watered growth condition modulates the expression of several genes involved in ROS signal perception, transduction, or response, which are known to be important for improving growth, ROS scavenging or sensitivity to stress (Fig. 8a). In addition, many genes related to PETC and antioxidant defenses are also differentially expressed in this type of plant, pointing toward a better protection of the photosynthetic apparatus against basal oxidative stress.

### Modulation of gene expression in OEX plants during drought conditions

Gene expression analysis by qRT-PCR revealed that several DEGs identified in well-watered OEX plants are also differentially expressed during drought (Fig. 7b, d–m), suggesting that they also play an important role in stress response pathways and chloroplast protection against ROS during drought as described under well-watered growth conditions (Fig. 8). However, analysis of other DEGs identified in OEX plants after drought treatment gives an insight on the function of additional genes regulated by TaZFP13D only during drought.

#### Improved ROS scavenging and down-regulation of stress markers

Overexpression of transcriptional regulators which confer stress tolerance often triggers the expression of stress-responsive genes. However, a tolerant phenotype clearly indicates that plants cope better with stress and this should be reflected by a reduction of several stress markers (Fig. 8b). Accordingly, we found that several HSPs (#149, #155, #164, #171, #197–198, #203, #555) are not as up-regulated by drought in OEX plants as they are in control plants (Online Resource 2, Figs. 7m and 8a). This regulation pattern is not surprising since HSPs expression is known to be increased in response to chloroplastic ROS production through the redox-regulated HSFs transcription factors (Foyer and Noctor 2005; Hu et al. 2020), and TaZFP13D OEX plants have improved ROS scavenging (Figs. 4 and 5) and reduced ROS accumulation in leaves (Bouard and Houde 2022).

#### Maintenance of rubisco activity

Rubisco activity is crucial to sustain PETC products consumption, which is important to avoid its over-reduction and ROS production. Interestingly, OEX plants show several up-regulated RBCS genes (#533, #535) coding for Rubisco small subunits (Online Resource 2, Fig. 8b). Previous work showed that RBCSs are important for Rubisco accumulation (Izumi et al. 2012). An increase in RBCS transcripts and concomitant increase in Rubisco level and activity may contribute to the drought resistance of OEX plants (Fig. 8b). Rubisco is a complex protein that is naturally inhibited by sugar phosphates, and removal of these sugars by RCA is required for the enzyme to remain in an active state (Parry et al. 2008, 2013). Rubisco and RCA are important actors in photosynthesis limitation in wheat plants exposed to drought stress (Perdomo et al. 2017). Accordingly, we found an up-regulated gene coding for Rubisco activase (RCA) (#534) in drought-treated OEX plants (Online Resource 2, Figs. 7n and 8b), which could help maintain Rubisco activity during water stress, avoiding PETC over-reduction and ROS production (Fig. 8b).

#### Adaptation to low water potential

We showed that OEX plants have enhanced resistance to drought-induced wilting (Fig. 3a). Interestingly, drought-treated OEX plants have an up-regulated gene coding for stomatal closure-related actin-binding protein 1 (SCAB1) (#532). In Arabidopsis, SCAB1 stabilizes and bundles actin filament in guard cells, which delays their disassembling and re-organization from radial to longitudinal orientation during stomatal closure (Li et al. 2022; Zhao et al. 2011). However, SCAB1 can also be important to regulate water loss during drought in OEX plants by maintaining stomata in a closed position through actin bundles stabilization in a longitudinal orientation (Fig. 8a). We also identified an up-regulated β-expansin11 (EXPB11) gene in OEX plants during drought stress (Online Resource 2, #538, Fig. 8a). Previous work showed that overexpression of wheat β-expansin23 in tobacco improves drought tolerance probably by increasing cell-wall elasticity (Li et al. 2011, 2013), which is important for cell turgor and growth maintenance under drought conditions (Le Gall et al. 2015). These results suggests that TaZFP13D is important to adapt cellular morphology to low water potential (Fig. 8a).

### TaZFP13D and TaZFP1B mostly regulate distinct gene subsets

The previously characterized TaZFP1B transcription factor was shown to positively regulate growth and drought tolerance in wheat, likely because it regulates many stress-related genes and genes that improve drought tolerance, especially following drought treatment (Cheuk et al. 2020). Interestingly, plants that over- or underexpress *TaZFP1B* and *TaZFP13D* show closely related phenotypes. It was therefore of interest to determine if these two genes regulate the same gene subsets in well-watered and drought conditions. Since DEGs regulated by TaZFP1B were previously identified with different tools, we have re-analyzed the TaZFP1B dataset (GEO repository, #GSE136683) with the approach used for TaZFP13D. Overall, 643 and 919 DEGs were identified in *TaZFP1B* OEX and siRNA plants, respectively (Online Resource 4). As expected, all the DEGs whose expression was previously validated by qRT-PCR (Cheuk et al. 2020) were retrieved in the corresponding cluster, which gives good confidence about the current DEGs selection method. Comparison between all the DEGs regulated by TaZFP1B and TaZFP13D showed that only 2.4% are commonly regulated by these two TaZFPs, indicating that they mostly regulate different gene subsets (Online Resource 5).

## Conclusions

This study demonstrates that TaZFP13D improves growth, ROS management and drought tolerance in wheat. Transcriptome profile analysis under well-watered growth conditions revealed that TaZFP13D regulates the expression of many genes associated with oxidative stress and drought tolerance, especially genes related to PETC, one of the main ROS producers during photosynthesis. The high number of DEGs related to PETC gives good confidence that TaZFP13D acts on this biological process to improve plant performance through water use efficiency and oxidative stress protection. The functions of these DEGs are in agreement with the positive effect of TaZFP13D on biomass production, ROS scavenging and drought tolerance. Thus, TaZFP13D and the previously characterized TaZFP1B (Cheuk et al. 2020) are both essential for drought tolerance. Despite the fact that TaZFP1B and TaZFP13D share several similarities like subcellular localization (Fig. 1) and expression profile (Cheuk and Houde 2016), they also show many differences like specific protein domains (Bouard and Houde 2022) and distinct gene subsets regulation (Online Resource 5) (Cheuk et al. 2020). Future work should aim to co-overexpress TaZFP1B and TaZFP13D to analyze their combined effect on drought tolerance. Both genes could be promising markers to develop new drought-tolerant wheat cultivars.

### Supplementary Information

Below is the link to the electronic supplementary material.

Supplementary material 1 (XLSX 12.8 kb)

Supplementary material 2 (XLSX 126.4 kb)

Supplementary material 3 (DOCX 81 kb)

Supplementary material 4 (XLSX 182.4 kb)

Supplementary material 5 (DOCX 53.1 kb)

Supplementary material 6 (DOCX 172.8 kb)

## Data Availability

The RNA-Seq datasets generated and/or analyzed during the current study are available in the GEO repository under the accession numbers: #GSE226842 (https://www.ncbi.nlm.nih.gov/geo/query/acc.cgi?acc=GSE226842) and #GSE136683 (https://www.ncbi.nlm.nih.gov/geo/query/acc.cgi?acc=GSE136683). The TaZFP13D sequence is available at Genbank under accession number OM630429 (https://www.ncbi.nlm.nih.gov/nuccore/OM630429).
