# Supplementary material for: Modulation of the wheat transcriptome by TaZFP13D under well-watered and drought conditions
Source: Plant Mol Biol. 2024 Feb 9;114(1):16. doi: 10.1007/s11103-023-01403-y (PMC10853348; doi:10.1007/s11103-023-01403-y)
Supplement: Supplementary file 3 — Supplementary material 3 (DOCX 81 kb) [file 11103_2023_1403_MOESM3_ESM.docx]

Modulation of the wheat transcriptome by TaZFP13D under well-watered and drought conditions

Plant Molecular Biology

William Bouard, François Ouellet, Mario Houde

houde.mario@uqam.ca

**
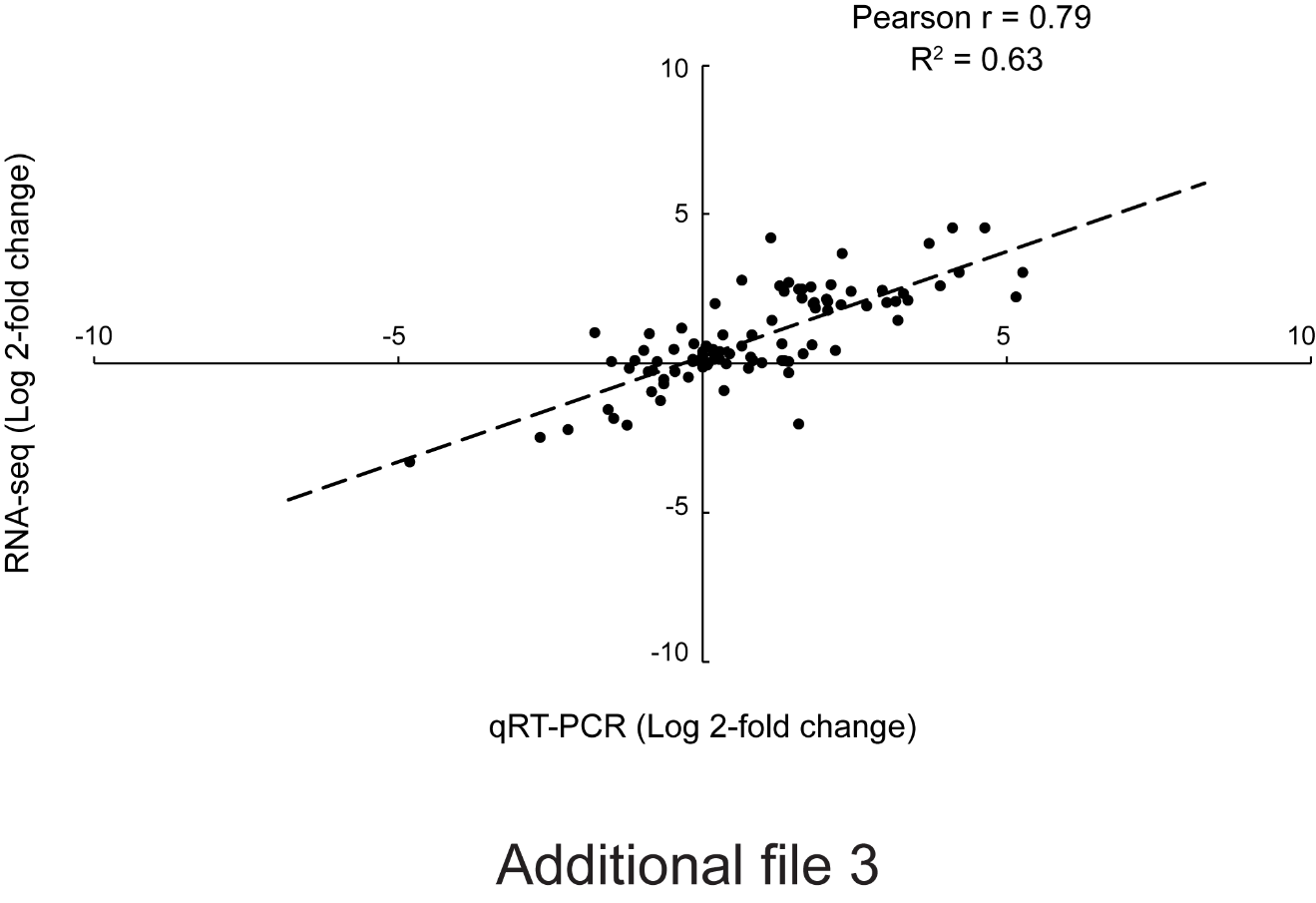
**

**Online resource 3: Pearson correlation between RNA-Seq data and qRT-PCR analysis of gene expression**

Log 2-fold change values of gene expression obtained from RNA-Seq data using the DESeq2 software were plotted against values obtained from qRT-PCR analysis (three different biological replicates). For both RNA-Seq and qRT-PCR data analysis, well-watered WT plants were used to normalize gene expression. This correlation analysis was done with the genes presented in Fig. 7. These values were used to calculate the Pearson correlation coefficient (Pearson r) and the coefficient of determination (R^2^).
