# Supplementary material for: Modulation of the wheat transcriptome by TaZFP13D under well-watered and drought conditions
Source: Plant Mol Biol. 2024 Feb 9;114(1):16. doi: 10.1007/s11103-023-01403-y (PMC10853348; doi:10.1007/s11103-023-01403-y)
Supplement: Supplementary file 5 — Supplementary material 5 (DOCX 53.1 kb) [file 11103_2023_1403_MOESM5_ESM.docx]

Modulation of the wheat transcriptome by TaZFP13D under well-watered and drought conditions

Plant Molecular Biology

William Bouard, François Ouellet, Mario Houde

houde.mario@uqam.ca


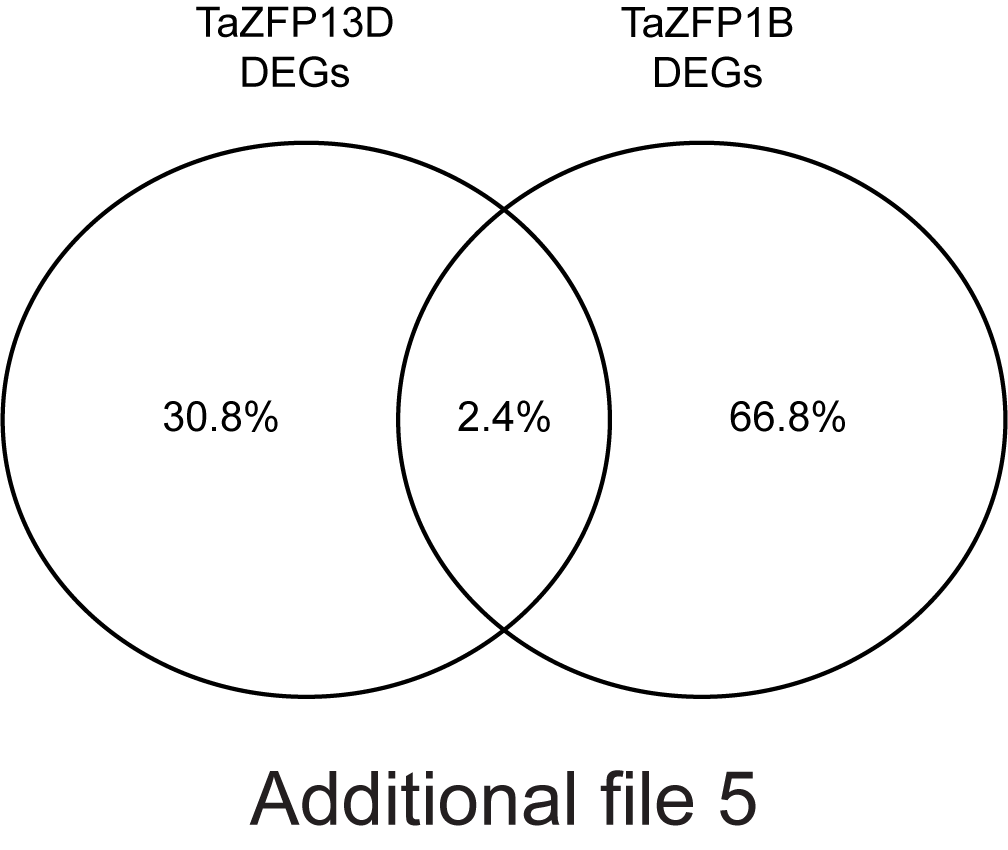


**Online Resource 5: The TaZFP13D regulon differs from the TaZFP1B regulon**

Venn diagram showing the comparison of the DEGs regulated by TaZFP13D (referenced in Online Resource 2) and TaZFP1B (referenced in Online Resource 4).
