# Supplementary material for: Modulation of the wheat transcriptome by TaZFP13D under well-watered and drought conditions
Source: Plant Mol Biol. 2024 Feb 9;114(1):16. doi: 10.1007/s11103-023-01403-y (PMC10853348; doi:10.1007/s11103-023-01403-y)
Supplement: Supplementary file 6 — Supplementary material 6 (DOCX 172.8 kb) [file 11103_2023_1403_MOESM6_ESM.docx]

Modulation of the wheat transcriptome by TaZFP13D under well-watered and drought conditions

Plant Molecular Biology

William Bouard, François Ouellet, Mario Houde

houde.mario@uqam.ca

**
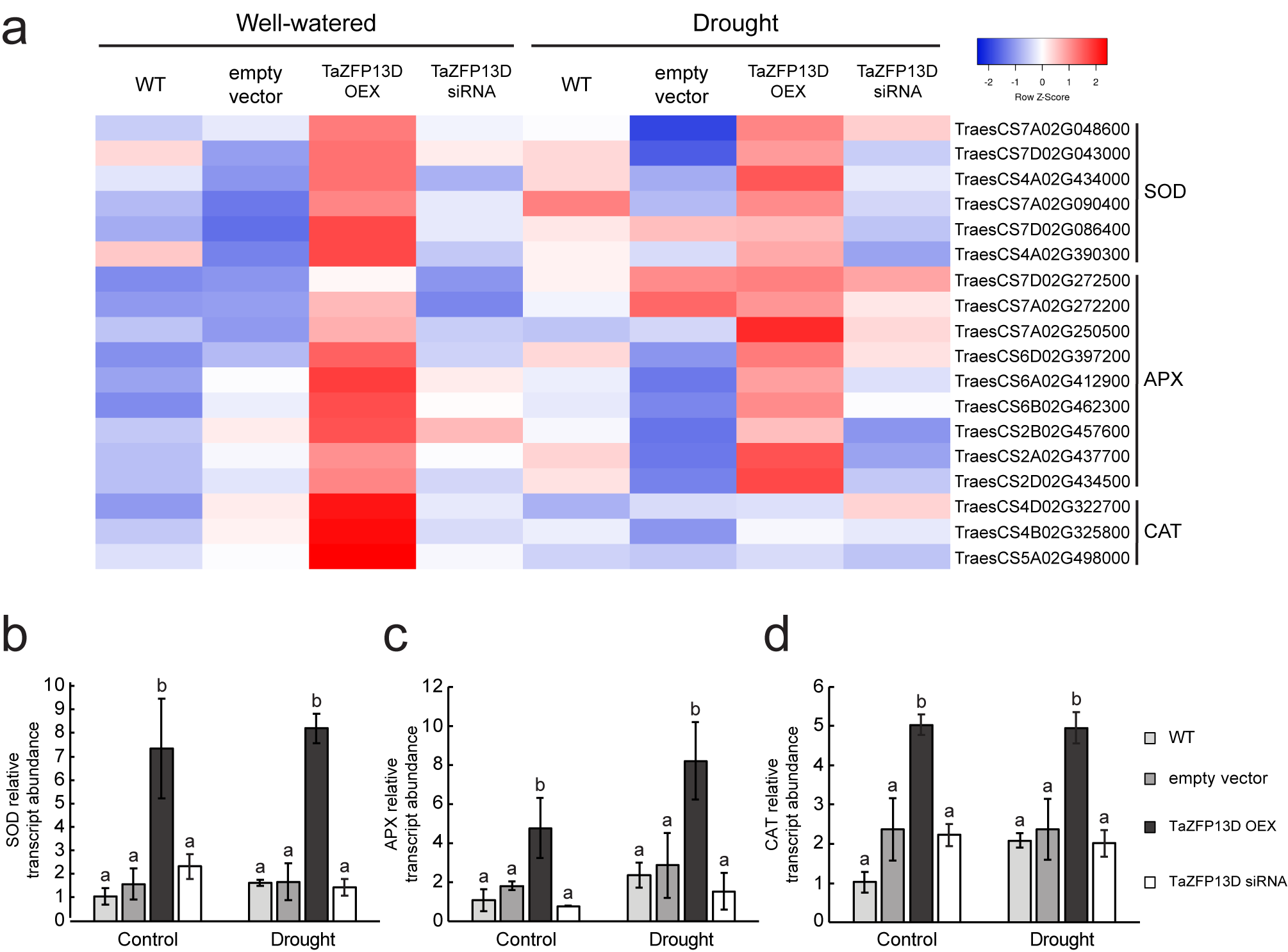
**

**Online Resource 6: *TaZFP13D* overexpression increases SOD, CAT and APX gene expression.**

Genes coding for superoxide dismutase (SOD), ascorbate peroxidase (APX) and catalase (CAT) were retrieved in the RNA-Seq data generated from Wild-type, empty vector, *TaZFP13D* OEX and *TaZFP13D* siRNA plants grown under well-watered conditions for 21 days (Control) or for 14 days before withholding water for 7 days (Drought). **(a)** Heatmap showing the expression of the selected DEGs in the different types of plants. **(b-d)** Expression levels of 8 genes encoding SOD **(b)**, APX **(c)** or CAT **(d)**, presented in (a), were validated by qRT-PCR. The high similarity between closely-related genes encoding each of these proteins did not allow for the design of specific primer pairs for a single gene, therefore two or three genes were measured concomitantly.
